# Rheological Assessment of Oil-Xanthan Emulsions in Terms of Complex, Storage, and Loss Moduli

**DOI:** 10.3390/polym15020470

**Published:** 2023-01-16

**Authors:** Mamdouh Taha Ghannam, Mohamed Y. E. Selim, Abdulrazag Y. Zekri, Nabil Esmail

**Affiliations:** 1 Department of Chemical and Petroleum Engineering, Faculty of Engineering, United Arab Emirates University, Al-Ain 15551, United Arab Emirates; 2 Department of Mechanical Engineering, Faculty of Engineering, United Arab Emirates University, Al-Ain 15551, United Arab Emirates; 3 Department of Mechanical Engineering, Concordia University, 1455 de Maisonneuve Boulevard W., Montreal, QC M3G 1M8, Canada

**Keywords:** complex modulus, storage modulus, loss modulus, crude oil, xanthan gums

## Abstract

This experimental assessment was carried out to study the viscoelastic performance of crude oil-xanthan emulsions employing a RheoStress RS100 rheometer. Crude oil with a concentration range of 0–75% by volume was used to prepare the oil-gum emulsions. Two xanthan gums of Sigma and Kelzan were added in the emulsions with concentration ranges of 0–10^4^ ppm. The linear viscoelastic ranges of all the tested oil-gum emulsions were found in the range of 0.1–10 Pa. Thus, the experimental tests were completed within the linear viscoelastic range of 1 Pa. The complex modulus increased gradually and steadily with frequency and gum concentration for all the examined emulsions. The addition of crude oil into the lighter xanthan concentration of <10^3^ ppm provided almost the same behavior as the xanthan solution, whereas the presence of crude oil within the higher xanthan concentrations significantly stimulated the measured values of the complex modulus. For lower gum concentrations of up to 1000 ppm, oil concentration displayed no effect on both the storage and loss moduli, whereas for gum concentrations higher than 1000 ppm, both moduli increased gradually with crude oil concentration.

## 1. Introduction

The application of polymer solutions in the enhanced oil recovery (EOR) stage is very helpful to preserve the crude oil production rate at the economical level after traditional techniques have been completed. As explained by Arjmand et al. (2012), Wang et al. (2013), and Wei et al. (2018), the pumping of polymer aqueous solutions into an oil formation is very efficient to acquire a substantial quantity of retained oil [1,2,3]. Therefore, the injection of polymer solutions into oil wells can be utilized effectively to extract more oil via raising the viscosity of the injected solution [4]. Polymers are employed in EOR as thickeners to modify the rheological characteristics of the aqueous phase to improve the mobility ratio and sweep efficiency and to escalate recovery and oil production rates [5]. This situation causes the development of a crude oil-polymer emulsion, i.e., the occurrence of distributed oil droplet phase within the continuous immiscible phase [6]. It is important to assess oil droplet phase involvement within the aqueous continuous phase to understand the oil displacement mechanism of polymer solutions.

Many experimental investigations have been carried out on the rheological measurements of emulsions in which the continuous phase is Newtonian fluid [7,8,9]. For a low concentration of oil phase within an aqueous phase, viscosity rises linearly with oil concentration. For a medium oil concentration, viscosity increases nonlinearly, while the emulsion is still Newtonian. However, at high oil concentrations, emulsions show non-Newtonian pseudoplastic behavior with associated yield stress at oil concentrations higher than 75% [10,11,12]. A lesser number of studies can be found on experimental rheological investigations of oil emulsion where a non-Newtonian polymeric solution is used as a continuous phase [6,13,14]. Sosa-Herrera et al. examined the rheological characteristics of an oil-water emulsion in which 30% sunflower oil and different sodium caseinate and gellan gum amounts were added [15]. They found low viscosity values and almost Newtonian behavior for caseinate, gellan gum, and mixed caseinate and gellan aqueous solutions. In addition, they noticed that the emulsions without gellan exhibited almost Newtonian behavior, whereas in the presence of 0.03% or higher gellan within an emulsion, the non-Newtonian behavior of shear thinning response was detected. Numerous experimental studies are available on the rheological behavior of dispersed solid particles in liquid emulsions [16,17,18,19,20].

The polysaccharide biopolymer xanthan gum is considered to be the most commonly used polymer in the flooding stage. Kamal et al. (2015) and Agi et al. (2018) each reported that many biopolymers could be utilized as injected polymers in EOR, but xanthan gum is the recommended biopolymer often utilized. It is stable at high temperature and salinity, reasonable in cost, non-poisonous, and environmentally friendly [21,22]. Consequently, xanthan gum delivers various positive features, such as forming aqueous solutions of required viscosity with smaller amounts of xanthan addition and being chemically stable for function in oil well conditions [23]. Xanthan gum is a high-molecular-weight polysaccharide formed by bacteria of the genus Xanthomonas campestris. There are many important applications that include xanthan gum, including the foodstuff, cosmetic, medicine, and oil industries [24]. Chemical and physical techniques have been used to study the characteristics of xanthan solutions [25,26]. Xanthan aqueous solutions show pseudoplastic and suspending activities. This kind of flow response allows xanthan to be included in a range of useful uses, including suspension, coagulation and emulsification agents in a variety of industries, such as the medical, foods, and crude oil industries [27]. Rodd et al. (2000) investigated the flow characteristics of xanthan gum solutions [28], and Whistler et al. (1997) found pseudoplasticity behavior of xanthan gum solutions due to the formation of high-molecular-weight assemblies of stiff rod molecules [29]. Milas et al. (1979) reported that xanthan macromolecules in a well-organized helical arrangement stiffened a polysaccharide solution [30], and this led the xanthan solution to be one of the stiffest natural biopolymers [31]. Morris (1995) and Sato et al. (1984) each reported that xanthan molecules formed stiff rods at low molecular weights and stiff, wormlike coils at high molecular weights [32,33].

Study of the viscoelastic characteristics of crude oil-xanthan emulsions is useful to understand the flow behavior of these emulsions in the EOR phase. The viscoelastic behavior of crude oil-gum emulsions is important for many practical practices, such as those in the crude oil industry. The purpose of this investigation is to explore the viscous and elastic behaviors for different crude oil-xanthan emulsions. For the current study, oscillated stresses are applied on a prepared crude oil-xanthan emulsion and compared against time. The resulting deformation is observed and analyzed. Viscoelastic materials contain long-chain molecular structures. At a minimum energy level, these molecules merge and loop with each other to provide a complicated arrangement. When oscillated stresses are applied, these molecules stretch and surge the bond vector angles, and their energy states are elevated. Predicting and understanding crude oil-xanthan emulsion flow behavior is necessary for the selection, design, and operation of equipment used in the preparing and pumping processes. The current work focuses on studying viscoelastic behavior in terms of complex, storage, and loss moduli for crude oil-xanthan gum emulsions employing a RheoStress RS100 rheometer. A wide range of crude oil and xanthan gum concentrations are covered in the presence of two types of xanthan gums.

## 2. Materials and Methods

### 2.1. Materials

#### 2.1.1. Crude Oil

North Sea crude was supplied by Shell Canada Limited with a viscosity equal to 7.16 mPas at 40 °C, a density of 880.6 kg/m^3^ at 15 °C, and an API of 29.1 at 288 K. The crude oil had an acid number of 0.0012 Kg KOH/Kg, which was obtained through the ASTM D664 standard titration procedure. Crude oil-gum emulsions were organized from crude oil, xanthan gum aqueous solution, and surfactant material. Two crude oil concentrations of 25% and 75% by volume were added into the polymer solutions to prepare the crude oil-xanthan emulsions. These two concentrations of crude oil were designated to represent typical examples of low and high crude oil involvement within gum emulsions.

#### 2.1.2. Emulsifying Agent

A surfactant material was required to formulate the crude oil-gum emulsions. A surfactant material is usually added into the oil-aqueous phase system as an emulsifying agent to achieve two objectives. The first one is to decrease the interfacial tension between the oil phase and the aqueous solution phase, hence forming an emulsion system. The second objective is to stabilize the occurrence of the oil droplet phase within the aqueous continuous phase to prevent the coalescence mechanism between oil droplets [7]. Triton X-100 (Sigma-Aldrich Canada Ltd.,) was utilized as an emulsifying agent, with a specific gravity of 1.07 and a flash point of 113 °C.

#### 2.1.3. Xanthan Gums

Two kinds of xanthan gums were used for this study. The first xanthan type was of chemical grade (Sigma-Aldrich Canada Ltd., Oakville, ON L6H 6J8, Canada), with a product name and number of Sigma and G1253, respectively. The other type was of industrial grade (CP Kelco, Atlanta, GA 30339, USA), with a product name and number of Kelzan and 10040282, respectively. These two kinds of xanthan gums were acquired and used without any extra treatment. Both kinds were white-to-tan-colored powders, with a molecular weight of 241.112 g/mol and a molecular formula of C_8_H_14_C_l2_N_2_O_2_ utilized for non-food applications, such as thickening and rheology control agents. The tested gum solutions were prepared by adding the xanthan powder slightly into a quarter liter of warm distilled water until all the gum completely dissolved. This study examined a wide range of concentrations over the range of 0–10^4^ ppm for the two types of gum materials. An amount of 1 gm of formaldehyde was dissolved into the samples, which were stored at 4 °C to avoid bacterial growth.

### 2.2. Methods

#### 2.2.1. Rheometer

A Rheostress RS100 rheometer (Haake GmbH, Karlsruhe, Germany) was used to carry out all the required measurements at a room temperature of 22 °C using the oscillation (OSC) mode of the cone-plate sensor. A water bath was attached to the RS100 instrument to control the assigned temperature. The RS100 reheometer inspected the resulting deformation of the examined emulsions with a digital encoder, which processed one million impulses per revolution to allow the detection of small strain values. The RS100 operating system could be simply switched between different operating modes; it also assigned oscillating stress and frequency sweeps. The sensor devised of the RS100 comprised of a stainless-steel cone and plate of 35 mm in diameter, with a 0.137 mm gap at the cone tip and a 4° cone angle [34].

#### 2.2.2. Dynamic Test

To address the viscoelastic characteristics of the crude oil-xanthan emulsions, we utilized a dynamic test in which the applied stress oscillated in a sinusoidal time function within the controlled stress mode of the RS100 instrument, as shown in Equation (1) [35]. The effects and the outcomes of the applied oscillating stresses on a given material were studied.

*τ = τ*_*a*_*sin (w t)* (1)

where *τ* is the applied shear stress in Pa, *τ_a_* is the stress amplitude in Pa, and *w* is the frequency in s^−1^. Equation (2) introduces the complex modulus G* in Pa, which designated the sample total resistance for the imposed stress and can be given as follows [35]:

G* = τ_*a*_*/γ*_*o*_ (2)

where *γ_o_* is the strain amplitude in -. The complex modulus G* comprises both the storage or elastic modulus term *G′* and the viscous or loss modulus term *G″.* The elastic modulus term described the stress energy that could be stored through the experiment and could be recovered, while the other term of the viscous modulus signified the stress energy dissipated to initiate the flow that was permanently spent into shear heat. For only viscous behavior, the test results showed that the viscous modulus *G″* almost equaled the complex modulus G*. However, for purely elastic behavior, the results specified that the elastic modulus *G′* almost equaled the complex modulus.

## 3. Results and Discussion

A prior study on crude oil-xanthan emulsion flow behavior reported non-Newtonian shear-thinning behavior in which the shear stresses of these emulsions relied on shear rate, crude oil, and xanthan concentrations. The apparent viscosities of the examined crude oil emulsions were higher than their corresponding aqueous solutions. For lower crude oil concentrations of ≤25% and gum concentrations of ≤1000 ppm, crude oil-gum emulsions displayed analogous viscosity behaviors, regardless of the gum type. The Casson model shown in Equation (3) could be utilized to predict the experimental measurements of crude oil-xanthan emulsions over the examined concentration range [36]. The xanthan solutions exhibited shear-thinning behavior in which the viscosity weakened with shear rate. Similar profiles were observed and reported by both Yeon et al. (2014) and Khan et al. (2018) [37,38]. Jha et al. (2018 and 2021) evaluated the influence of polymers on the rheological characteristics of oil-water emulsions and the consequences of xanthan gum on drilling fluids. These studies concluded significant shear-thinning profiles of the examined emulsions [39,40].

(3) $$\tau \;=\;{\left[{\tau_{o}}^{0.5}\;+\;{\left(\dot{\gamma }\eta_{c}\right)}^{0.5}\right]}^{2}$$

where *τ* is the shear stress in Pa, *τ_o_* is the apparent yield stress in Pa, $\dot{\gamma }$ is the shear rate in s^−1^, and *η_c_* is the Casson apparent viscosity in Pa.s.

It is important to utilize the stress sweep test on crude oil-xanthan emulsions to determine the linear viscoelastic range. This is the range where the complex modulus remains unaffected with the applied stress. This examination excluded considering the viscoelastic features of emulsions within the non-linear viscoelastic range. Within the non-linear viscoelastic range, the sample structure is stressed considerably to the level that the temporary bonds are demolished, and a significant contribution of the introduced energy disappears as heat [35]. Different crude oil-xanthan emulsions of low, medium, and high concentrations were examined through stress sweep tests over the applied stress range of 0.1–100 Pa. The results showed that the linear viscoelastic ranges for all the tested emulsions were found in the range of 0.1–10 Pa. Accordingly, all the following measurements were carried out at the applied stress of 1 Pa to confirm that the crude oil emulsion samples were tested within the linear viscoelastic range.

### 3.1. Complex Modulus Behavior of the Crude Oil-Xanthan Emulsions

**Figure 1:**
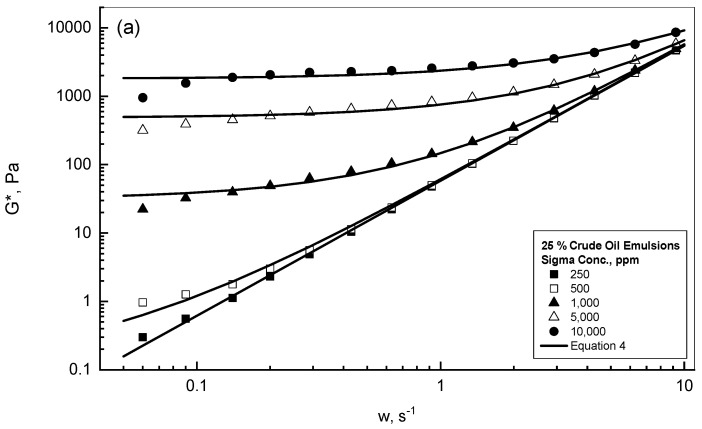

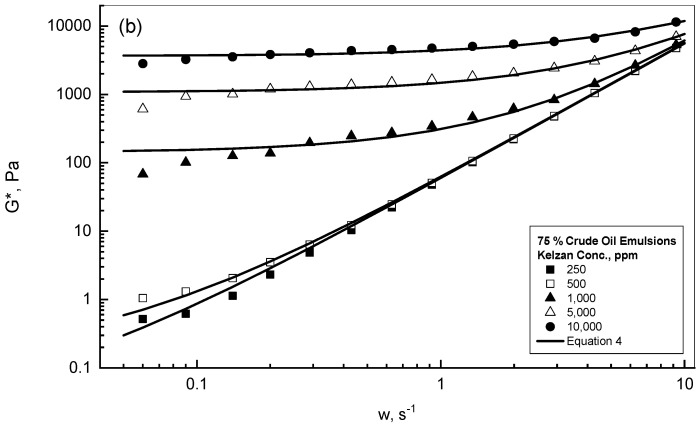
G* different behaviors of oil-gum emulsions. (**a**) 25% crude oil emulsions; (**b**) 75% crude oil emulsions.

The complex modulus G* of the different emulsions was investigated against the oscillatory shear influence in the frequency range of 0.05–10 s^−1^. Figure 1a shows the complex modulus G* behavior of 25% crude oil-Sigma emulsions in the presence of different concentrations of Sigma material over a range of 250–10^4^ ppm, while Figure 1b displays a similar study but for 75% crude oil-Kelzan emulsions over the same frequency range. The complex modulus G* increased gradually and steadily with frequency for all the examined emulsions, as can be noted from both Figure 1a,b. For lower gum concentrations of up to 500 ppm, both Figure 1a,b display almost similar and linear behavior of the G* with frequency. Both figures exhibit a slight influence of xanthan concentration on the complex modulus G* up to the frequency of 1 s^−1^. Outside the reported frequency range of 1 s^−1^, the concentration effect totally disappeared, and both curves combined together into one linear behavior depending solely on frequency up to 10 s^−1^. However, for the concentrations higher than 500 ppm, gum concentration strongly dictated the complex modulus behavior over the whole range of the applied frequency, with a nearly linear contour followed by non-linear behavior at a certain value of critical frequency *w_c_*. This critical frequency increased with gum concentration, as given in Table 1. All the reported curves of both figures approached each other and formed one master curve, as can be observed from this investigation. Aomari et al. (1998) reported similar behavior for an investigation of sea water-crude oil emulsions in which the loss modulus showed a maximum value at a certain shear strain; however, such shear strain decreased as the volume fraction of the dispersed phase increased [41]. 

At this stage, it is useful to address the influence of gum concentration and frequency. For the lighter concentration group (i.e., less than 10^3^ ppm), the rate of change of the complex modulus with frequency was almost constant, regardless of the gum concentration. For the heavier concentration group (i.e., ≥10^3^ ppm), the G* increased linearly up to a critical frequency value of *w_c_*, which relied upon the polymer concentration. For the applied frequencies higher than *w_c_*, more deformation rates affected the three-dimensional network emulsions structure, which resulted in larger G* values. The complex modulus G* presented the sample total resistance due to the effect of the assigned stress, which increased significantly with xanthan concentration, as can be concluded from Figure 1 and Table 2. Therefore, the deformation of the examined crude oil-xanthan emulsions was more difficult with higher polymer concentrations, as has also been concluded by Li et al. (2002) and Dolz et al. (2008) [42,43]. 

A detailed modeling analysis study for all the examined crude oil-xanthan emulsions was carried out to find a suitable mathematical model that could be utilized to describe the behavior reported in Figure 1. Consequently, the modeling analysis concluded that Equation (4) was sufficiently acceptable to describe the complex modulus behavior versus the assigned frequency, as can be observed from the solid curves in Figure 1. The *G_o_* defined the apparent complex modulus at zero assigned frequency. Table 2 displays all the fitting results according to Equation (4) for all the Sigma and Kelzan crude oil emulsions and the regression coefficient r^2^. Equation (4), as can be realized from Figure 1a,b, predicts very well the complex modulus behavior for all the tested emulsions. Table 2 reports the same results that were stated earlier, i.e., the 10^3^ ppm xanthan concentration was the border concentration for all the examined emulsions. For all the examined emulsions with xanthan concentrations less than 10^3^ ppm, Table 2 exhibits minor values with slight changes in *G_o_* and a power index *n* of almost 2. For the other concentrations of≥10^3^ ppm, Table 2 displays much higher values of *G_o_* with xanthan addition.

G* = [*G*_*o*_^0.5^
*+ B*^0.5^
*w*]^*n*^ (4)

**Figure 2:**
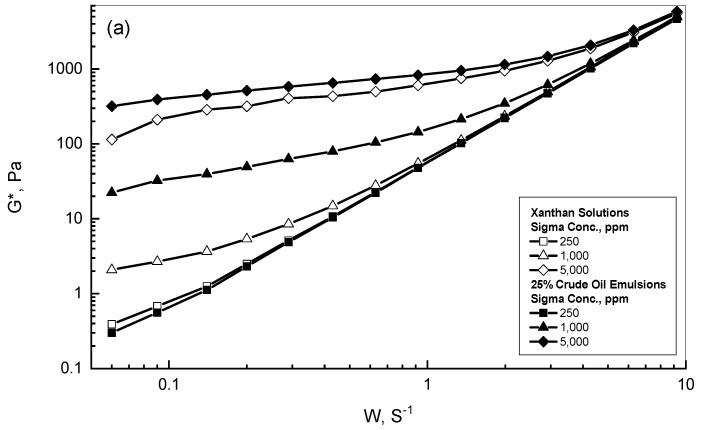

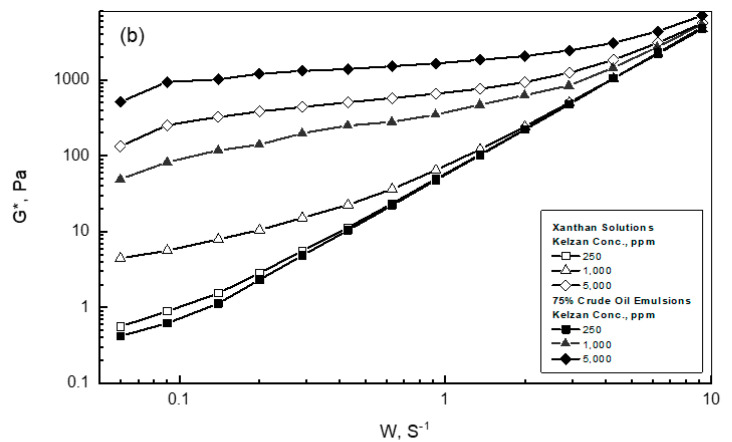
G* different behaviors of xanthan solutions and emulsions. (**a**) 25% crude oil emulsions; (**b**) 75% crude oil emulsions.

To explore the role of the crude oil within the crude oil-xanthan emulsions, the complex modulus G* of the selected xanthan solutions with concentration ranges of 250–5000 ppm and their corresponding emulsions were experimentally studied using oscillatory shear effect over the range of 0.05–10 s^−1^. Figure 2a,b illustrate complex modulus behavior versus frequency for different concentrations of Sigma and Kelzan, respectively. The reported experimental values of the complex modulus increased steadily and gradually with frequency for all the examined Sigma and Kelzan solutions. For the lower concentrations of Sigma and Kelzan, the G* varied almost linearly with the applied frequency, as can be concluded from Figure 2a,b. On the other hand, for the higher concentrations of 1000 and 5000 ppm, the reported G* behavior exhibited non-linear behavior that was different from that described for the lighter concentration. Both Figure 2a,b display the significant role of xanthan concentration at low frequencies, which vanished gradually with increasing frequency to the level that all the reported curves approached each other and developed into nearly one curve at a frequency of 10 s^−1^. The addition of the crude oil into the lighter xanthan concentration provided almost the same behavior for the xanthan aqueous solutions. However, as seen in Figure 2a,b, the existence of the crude oil within the higher xanthan concentrations of 10^3^ and 5000 ppm significantly promoted the measured values of the complex modulus due to the generated interactions between the crude oil droplet phase and the three-dimensional network structure of the xanthan gum. As has been concluded by Ghannam (2003) and Ghannam et al. (2014), the addition of crude oil into the polymer solution enhanced the dynamic viscosity of the prepared emulsions due to droplet interaction and collision with the formed polymer networks [6,36]. 

**Figure 3:**
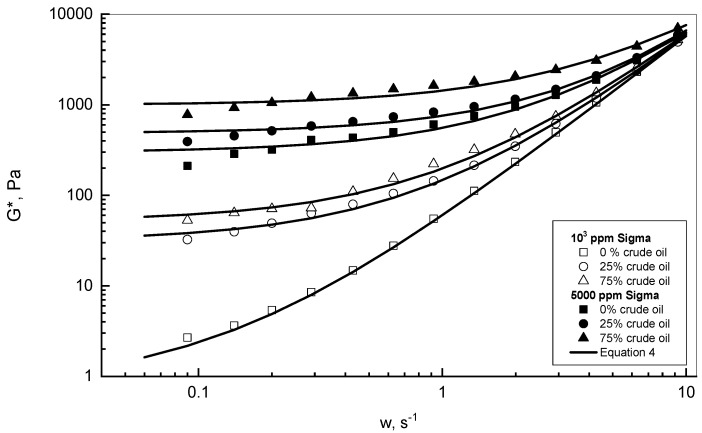
Effect of oil addition on G* behavior.

Figure 3 emphasizes the role of the crude oil addition into two different concentrations of Sigma solutions. The measured complex modulus increased gradually with crude oil addition over the range of 0–75% by volume of crude oil. For the 10^3^ ppm Sigma solution, the addition of 25% crude oil increased the measured G* considerably in comparison with its solutions. The greater addition of 75% crude oil slightly increased the measured G*. Similar observations are presented in Figure 3 for the 5000 ppm Sigma solution. As can be noticed from Figure 3, the addition of the crude oil droplet phase significantly boosted the complex modulus. The influence of the crude oil addition could be due to the interactions of the oil droplet distributed phase and the xanthan continuous phase. The greater the amount of crude oil involved, the higher the result for G*. The presence of the dispersed crude oil phase within the xanthan gum continuous phase improved the resistance of the emulsion deformation, which could be generated from the strong interactions and collisions between the oil phase and the polymer network [6,36]. The complex modulus behavior of the crude oil-xanthan emulsions was in consent with the features of the flow behavior discussed earlier due to the existence of the oil phase [36]. Sosa-Herrera et al. (2008) found that the elastic contribution of oil emulsions was higher than that of polymer solutions due to the occurrence of the distributed oil droplets within the polymer solution [15,44]. Furthermore, higher addition of the dissolved xanthan materials resulted in higher G* measurements, as can be found in Figure 3. Therefore, Figure 3 displays the enhancement role for the presence of crude oil within the xanthan solutions on the measured values of G*. As can be detected from Figure 3, the three-parameter model of Equation 4 fit the behavior of G* versus w with an r^2^ in excess of 0.99, which displayed a very good agreement between the experimental measurements and their corresponding predictions obtained by Equation (4), except at the very low frequency range. The solid lines in Figure 3 are the plots of the predicted values obtained from Equation (4), which show good agreement with the experimental measurements.

**Figure 4:**
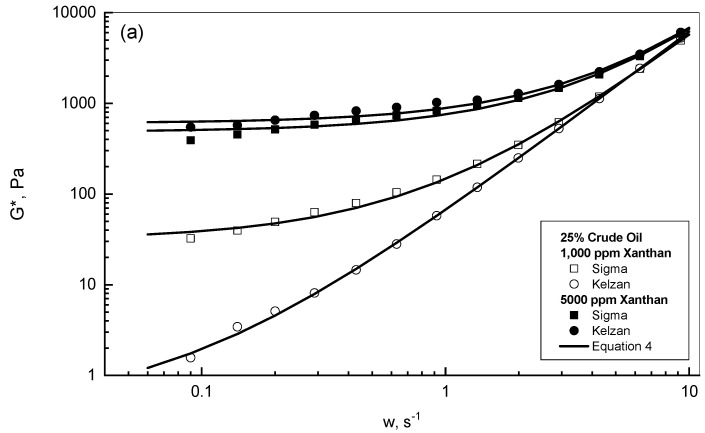

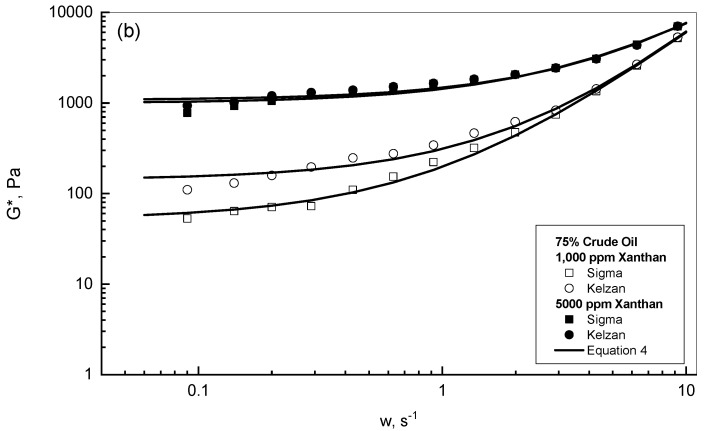
G* behavior of Sigma and Kelzan emulsions. (**a**) 25% crude oil emulsions; (**b**) 75% crude oil emulsions.

The dynamic test behavior of the two examined xanthan emulsions of Sigma and Kelzan in terms of complex modulus G* against frequency for the 25% and 75% crude oil concentrations are demonstrated in Figure 4a,b. Two xanthan concentrations of 1000 and 5000 ppm are displayed as typical examples. For the lower concentrations of 1000 ppm xanthan and 25% crude oil, this test revealed that the complex modulus values of Sigma were significantly higher than the reported values of the Kelzan emulsion. However, this discrepancy between the Sigma and Kelzan behaviors declined with the greater addition of crude oil at 75%. For the higher xanthan concentrations of 5000 ppm, both Figure 4a,b show almost similar and close behaviors of Sigma and Kelzan for the 25% and 75% crude oil emulsions. Figure 4a,b display the prediction results acquired from Equation (4) in solid lines, which reflect the degree of closeness between the experimental and the prediction results.

**Figure 5:**
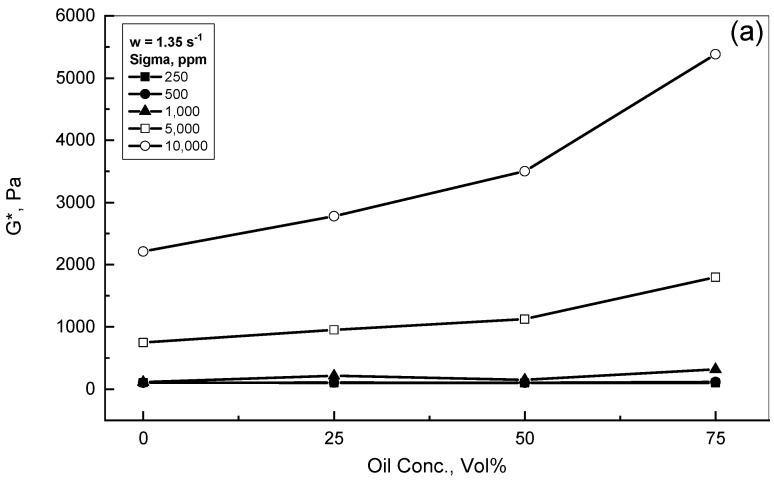

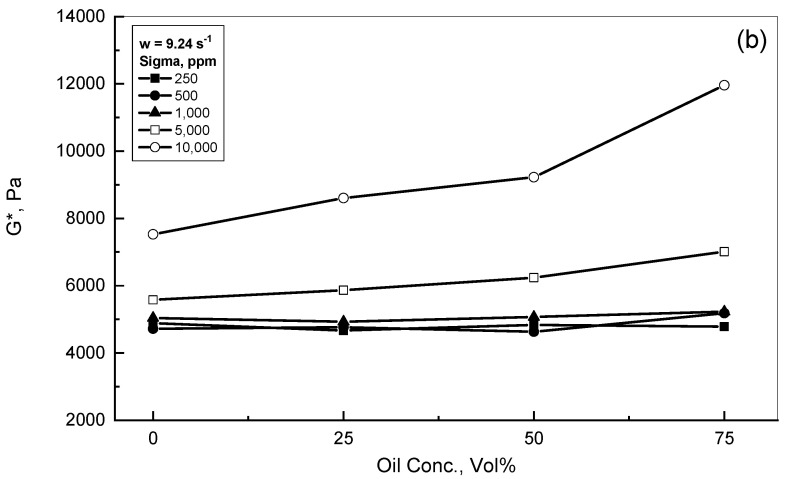
Effect of crude oil addition on G* behavior. (**a**) w = 1.35 s^−1^; (**b**) w = 9.24 s^−1^.

Figure 5a,b illustrate the effect of the crude oil concentrations on the complex modulus behavior for different Sigma emulsions. Two assigned frequency values of 1.35 s^−1^ and 9.24 s^−1^ were examined and are reported in Figure 5a,b, respectively. For the lower concentration Sigma emulsions with a concentration range of 250–1000 ppm, both Figures show almost similar and close profiles to each other, regardless of the concentrations of polymer and crude oil addition. On the other hand, the role of the crude oil addition was more significant in the presence of higher polymer concentrations, which is in a full agreement with the results obtained by Sosa-Herrera et al. (2008) [15]. Figure 5a,b show that the complex modulus rose steadily for the 5000 ppm Sigma concentration, and even more in the presence of 10^4^ ppm Sigma with increasing oil concentration. These results could be attributed to the contribution of the crude oil presence and the polymer network structures of the high Sigma concentrations. Therefore, the higher xanthan concentration was added, and much higher crude oil droplet interaction levels resulted, which eventually improved the complex modulus behavior.

### 3.2. Crude Oil-Xanthan Emulsion Viscoelastic Behavior in Terms of G′ and G″

**Figure 6:**
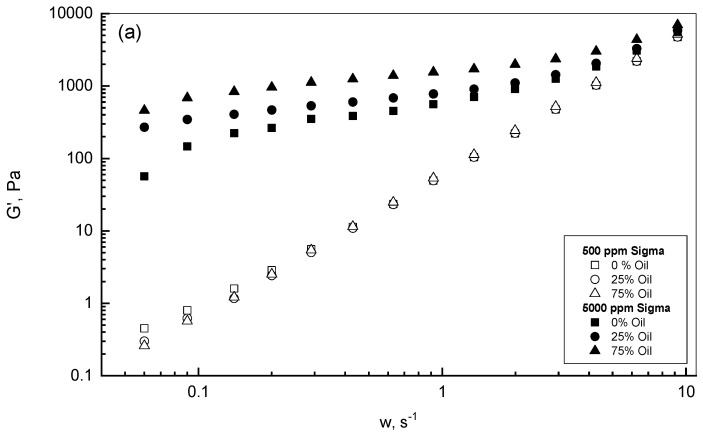

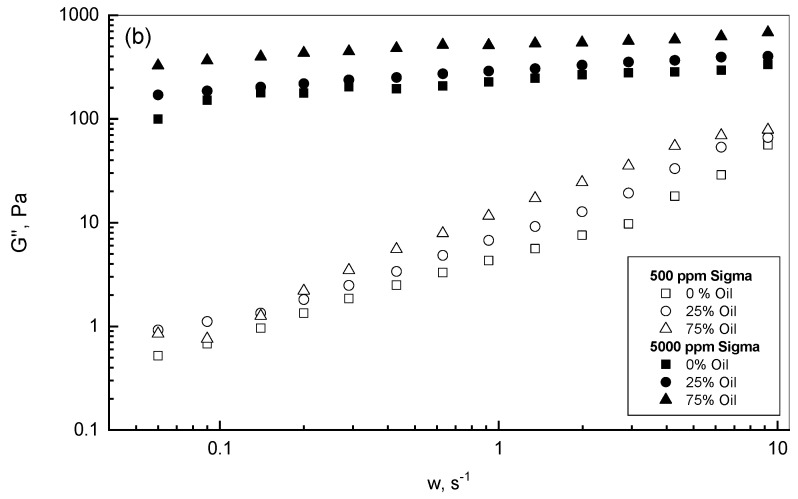
*G′* (**a**) and *G″* (**b**) behaviors of Sigma emulsions.

The viscoelastic behavior in terms of storage modulus *G′* and loss modulus *G″* is displayed in Figure 6a,b, respectively, for different Sigma emulsions to reveal the roles of xanthan and crude oil concentrations. Three frequency cycles from 0.05 to 10 s^−1^ were examined to study the xanthan emulsion viscoelastic behaviors on logarithmic scales to show the whole range of results for *G′* and *G″* versus w. For low xanthan concentrations of 500 ppm, Figure 6a shows independent behavior of the storage modulus *G′* on the oil concentration due to the low xanthan concentration and, consequently, insignificant oil droplet interactions. On the other hand, for the higher xanthan concentrations of 5000 ppm, the effect of the crude oil presence was more pronounced, especially at low frequencies. However, this discrepancy reduced with frequency to develop almost one curve at a frequency of 10 s^−1^. Figure 6b displays that the loss modulus *G″* increased gradually with frequency and slightly with oil concentration for low xanthan concentrations of 500 ppm, whereas this behavior was moderated in the presence of 5000 ppm xanthan. Both Figure 6a,b show that the values of *G′* and *G″* were significantly higher for 5000 ppm than their corresponding behaviors for 500 ppm. Therefore, the accumulation of xanthan concentration in the crude oil emulsions allowed the extension of the polymer chains and, therefore, the construction of entanglements and a stronger gel network.

**Figure 7:**
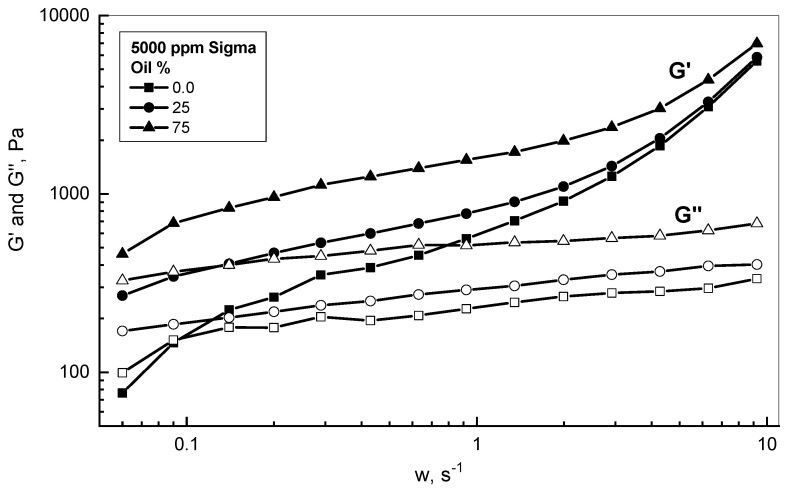
Viscoelastic behaviors of different 5000 ppm Sigma emulsions.

Figure 7 demonstrates a comparison of viscous and elastic behaviors in terms of *G′* and *G″* assessment. This study covered two different concentrations of low and high crude oil concentrations of 25% and 75%, respectively, in comparison with 5000 ppm Sigma solution. Some observations can be made from the comparison of the reported behaviors of *G′* and *G″* for the different examined emulsions. The Sigma solution exhibited a viscoelastic response with viscous behavior (i.e., liquid-like) at a frequency lower than 0.1 s^−1^ and elastic behavior (i.e., solid-like) at higher frequencies, which is typical for gel behavior. The crossover between the behavior of *G′* and *G″* occurred at 0.1 s^−1^. Secondly, the Sigma emulsions demonstrated mainly elastic behavior, as the storage modulus *G′* values were situated well above the loss modulus *G″* values for the whole range of the examined frequency. Therefore, the existence of crude oil droplets within the xanthan medium enhanced the elastic contribution of the examined emulsions, as concluded by Pal (1996) [45].

**Figure 8:**
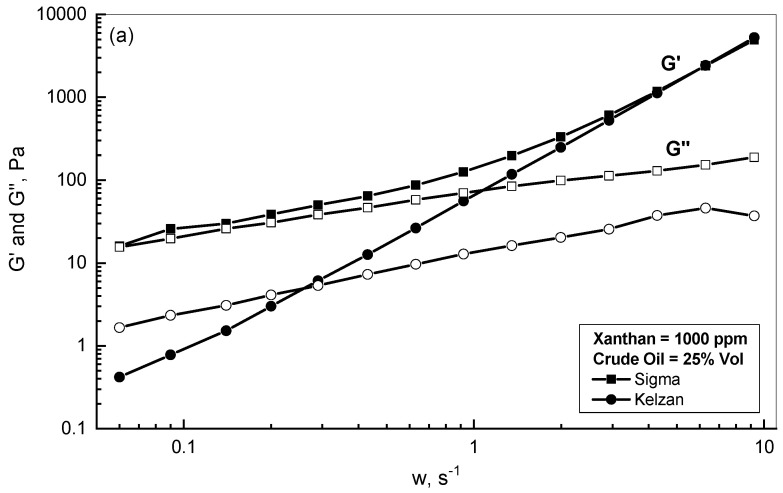

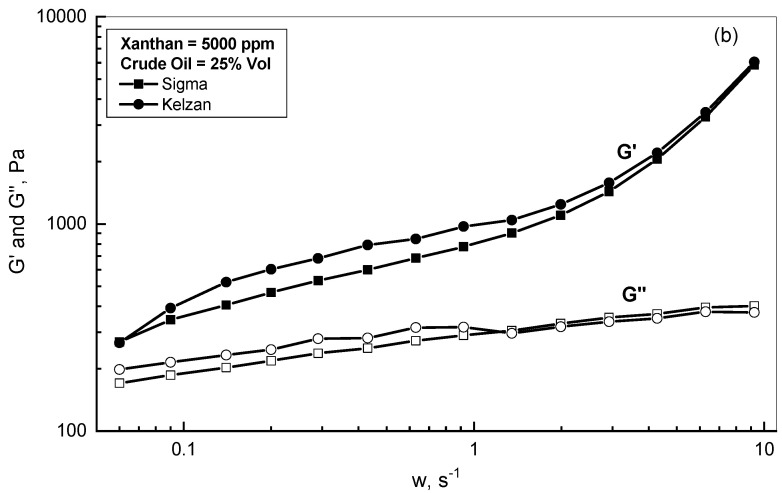
*G′* and *G″* behaviors of different Sigma and Kelzan emulsions. (**a**) Xanthan = 1000 ppm; (**b**) Xanthan = 5000 ppm.

Figure 8a,b display the profile behaviors in terms of *G′* and *G″* versus assigned frequency for the two examined xanthan emulsions of low and high polymer concentrations, respectively. For the xanthan concentration of 1000 ppm, Figure 8-a shows that both xanthan emulsions exhibited elastic behavior for frequencies higher than 0.3 s^−1^ since the *G′* for both emulsions were positioned above their *G″* curves. Furthermore, Figure 8-a displays that the 25% crude oil-Sigma emulsion resulted in higher *G′* and *G″* values than the corresponding values for the 25% crude oil-Kelzan emulsion up to the frequency of 3 s^−1^. For the higher xanthan concentration of 5000 ppm, Figure 8b shows that the emulsions of both Sigma and Kelzan illustrated similar profiles with mostly elastic behavior, as the storage modulus values were located well above the loss modulus for the whole range of the examined frequency.

**Figure 9:**
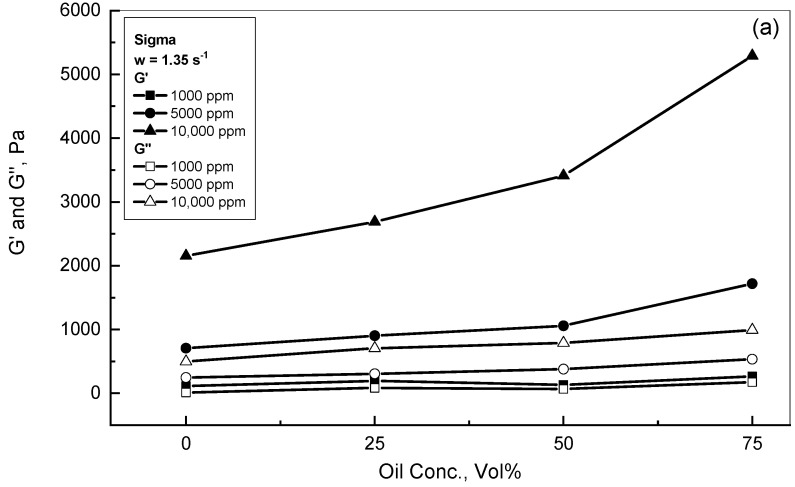

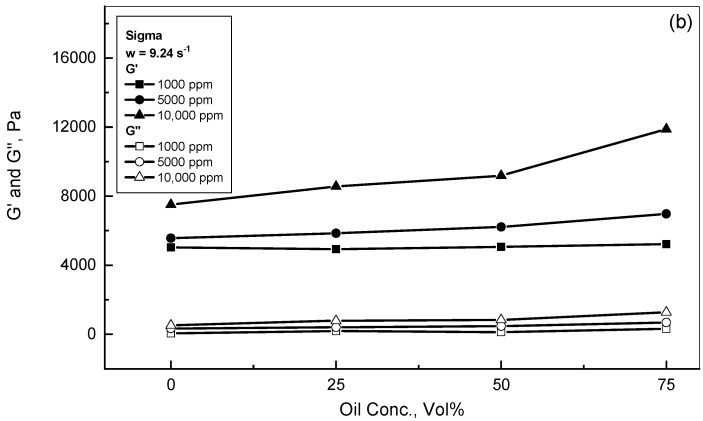
Effect of crude oil addition on the behavior of *G′* and *G″*. (**a**) w = 1.35 s^−1^; (**b**) w = 9.24 s^−1^.

Figure 9 displays the effect of crude oil concentration over the range of 0–75% by volume on the behavior of *G′* and *G″* for Sigma emulsions. Two different frequency values of 1.35 and 9.24 s^−1^, as typical examples of low and high frequencies, are presented in Figure 9a,b, respectively. Both Figures exhibit that all the tested emulsions provided significantly higher storage modulus values than their counterpart of loss modulus for each xanthan concentration. For the lower xanthan concentration of 1000 ppm, oil concentration revealed no effect on either *G′* or *G″* for the two cases of frequencies. However, for the xanthan concentrations higher than 1000 ppm, *G′* and *G″* grew gradually with the crude oil concentration. Nakamura et al. (2004) reported that the surface charges of xanthan molecules permitted for the interconnection for the oil and xanthan surfaces [46]. Many investigations have reported that hydrogen bonds and electrostatic interactions show strong influences on oil-gum structures [47]. Jiang et al. (2020) noted that the hydrogen bonding and electrostatic interactions of oil-gum emulsions developed pseudoplastic behaviors with oil elastic response [48]. Accordingly, these results for crude oil-xanthan emulsions were ascribed as follows: firstly, the existence of the high xanthan concentration continuous phase, which was viscoelastic in principle; secondly, an oil droplet distributed phase with high concentration, which displayed some degree of elastic behavior; and thirdly, the elasticity responses that were generated from the interactions between the oil droplets and the xanthan networks formed within the emulsions.

From the general observation of the current study, the crude oil-xanthan emulsions showed viscoelastic activities for all the studied xanthan types and crude oil concentrations. The crude oil and xanthan concentrations promoted the response of the complex modulus, which was the total resistance of the emulsions against the assigned dynamic shear. The variations in the elastic and viscous moduli under the effect of dynamic shear provided useful features to comprehend the rheological aspects of the crude oil-xanthan emulsions. Understanding the xanthan emulsion behavior through viscoelastic investigations under different conditions had a significant impact and offered important knowledge of the used polymer molecular structures, which facilitated the ability to change molecular structure to achieve special application needs. Once the assigned frequency exceeded the crossing point between the viscous and elastic profiles, the common concluded behavior was that *G′* significantly surpassed *G″*. This result reflected that the elastic activities dictated the overall behavior, which is usual for gel- and solid-like behaviors. Furthermore, this indicates that some sort of networks were structured within crude oil-xanthan emulsions [49].

## 4. Conclusions

A viscoelastic investigation of crude oil-xanthan emulsions in terms of complex, storage, and loss moduli was completed. All the tested emulsions exhibited linear viscoelastic behavior within the range of 0.1–10 Pa. For gum concentrations of≥10^3^ ppm, the gum addition displayed a strong effect on the complex modulus G* behavior in a linear profile, followed by non-linear behavior at a certain critical frequency. The results of the Sigma and Kelzan emulsions reported almost similar *w*_*c*_ values, which rose with gum concentration. The mathematical model of Equation (4) could be used to describe the complex modulus behavior. The existence of crude oil within the lower gum additions of <10^3^ ppm exhibited almost similar behavior to the gum solutions, while the occurrence of crude oil within the higher xanthan concentrations increased the measured values of G* considerably. For the lower concentrations of 1000 ppm xanthan and 25% crude oil, the G* values of Sigma were significantly higher than the reported values of the Kelzan emulsion. This divergence between the Sigma and Kelzan behaviors declined with the greater addition of crude oil. For the higher gum content of 5000 ppm, both Sigma and Kelzan illustrated comparable and adjacent behaviors for the 25% and 75% crude oil emulsions. For the lower gum contents of ≤10^3^ ppm, the G* displayed independent behavior from crude oil addition, whereas the role of crude oil addition was much more substantial in the presence of xanthan content >10^3^ ppm due to oil droplet interactions with the xanthan network structure. The storage modulus *G′* increased with oil addition in the presence of higher xanthan content due to strong interactions between the oil droplets and the xanthan network structure. For the xanthan content of≥1000 ppm, both the Sigma and Kelzan emulsions displayed mainly elastic behavior for frequencies higher than 0.3 s^−1^. For the higher xanthan concentration of 5000 ppm, both the Sigma and Kelzan emulsions exhibited similar profiles, with mostly elastic behavior. For the xanthan content of ≤10^3^ ppm, both *G′* and *G″* showed independent behavior from oil concentration. However, for xanthan concentrations of >10^3^ ppm, both moduli rose gradually with crude oil.

## Figures and Tables

**Table 1:** Crude oil-gum emulsion critical frequency *w_c_*.

Gum Conc., ppm	25% Crude Oil	75% Crude Oil
1000	0.2	0.3
5000	0.4	0.6
10,000	0.9	1.0

**Table 2:** Equation 4 fitting analysis.

**(a) 25% Crude Oil-Sigma Emulsions**				
**Sigma Conc., ppm**	***G_o_***	***B***	***n***	**r^2^**
250	0.0	61.415	1.973	0.999
500	0.12	56.117	1.998	0.999
10^3^	25.952	29.767	2.117	0.999
5000	204.866	8.781	2.325	0.997
10,000	16,884.92	587.493	1.541	0.975
**(b) 75% Crude Oil-Kelzan emulsions**				
**Kelzan Conc., ppm**	***G_o_***	***B***	***n***	**r^2^**
250	0.025	59.708	1.983	0.999
500	0.15	57.450	1.994	0.999
10^3^	65.988	10.116	2.366	0.999
5000	1359.302	41.616	1.936	0.984
10,000	1,068,503	31,003	1.182	0.968

## Data Availability

All research data used were reported and displayed in the current manuscript.
